# Sex-Related Difference in Outcomes of Remote Ischemic Conditioning for Symptomatic Intracranial Atherosclerotic Stenosis

**DOI:** 10.34133/cbsystems.0275

**Published:** 2025-06-06

**Authors:** Yuanyuan Liu, Chengbei Hou, Xiao Dong, Di Wu, Xuehong Chu, Jiaqi Luo, Wanwan Zhang, Erlan Yu, Chuanhui Li, Chen Zhou, Chuanjie Wu, Xunming Ji

**Affiliations:** ^1^ Department of Neurology, Xuanwu Hospital, Capital Medical University, Beijing, China.; ^2^ Center for Evidence Based Medicine, Xuanwu Hospital, Capital Medical University, Beijing, China.; ^3^ China-America Institute of Neuroscience and Beijing Institute of Geriatrics, Xuanwu Hospital, Capital Medical University, Beijing 100053, China.; ^4^ Beijing Institute for Brain Disorders, Capital Medical University, Beijing 100069, China.

## Abstract

Remote ischemic conditioning (RIC) is a novel and promising therapeutic intervention for symptomatic intracranial atherosclerotic stenosis (sICAS). This study aimed to evaluate sex differences in stroke recurrence among patients with sICAS and assess the efficacy of RIC in the RICA (chronic remote ischemic conditioning in patients with symptomatic intracranial atherosclerotic stenosis) trial. The RICA trial was a multicenter, randomized clinical trial conducted across 84 stroke centers in China. Patients with sICAS were randomly assigned on a 1:1 ratio to receive either RIC intervention or sham RIC intervention once daily for 12 months. The primary endpoint was ischemic stroke recurrence. The median follow-up duration was 3.5 years. Of the 3,033 patients enrolled in the RICA trial, 1,079 (35.58%) were women. Female patients were generally older (mean [SD] age 62.9 [8.8] years versus 60 [9.2] years) and had a higher prevalence of hypertension, diabetes, and a higher body mass index than male patients. No significant difference was observed in ischemic stroke recurrence risk between female and male patients during a median follow-up of 3.5 years (20.5% versus 16.6%, adjusted hazard ratio, 1.18; [95% CI, 0.97 to 1.42]). However, RIC significantly reduced the risk of ischemic stroke recurrence in male patients, while no similar effect was observed in female patients (adjusted hazard ratio, 0.88; [95% CI, 0.58 to 1.32]; *P* for interaction = 0.379). No significant sex-based differences were observed in ischemic stroke recurrence among patients with sICAS over the 3.5-year follow-up period. RIC may have better therapeutic benefits for male patients with good compliance.

## Introduction

Stroke is the main cause of death and disability [1,2]. Many studies have reported sex differences in stroke. Females experience a higher proportion of stroke-related deaths and disabilities, which may be due to factors such as atypical symptoms, delays in seeking care, sex-specific risks (e.g., pregnancy and hormonal contraceptive use), and the varied effects of modifiable risk factors [3–8]. Intracranial atherosclerotic stenosis (ICAS) is a predominant cause of stroke [9,10]. A high recurrence rate has been reported in symptomatic intracranial atherosclerotic stenosis (sICAS) patients. But the sex difference in ischemic stroke recurrence among sICAS patients remains uncertain. Recognizing potential sex differences in sICAS patients is crucial to ensure fair access and optimizing outcomes.

For sICAS patients, even after receiving aggressive pharmacologic therapy, patients with sICAS remain at high risk for stroke recurrence [9]. It is therefore essential that additional treatments be administered to reduce the rate of stroke recurrence among sICAS patients. Remote ischemic conditioning (RIC) is a simple intervention that can produce repetitive transient ischemic of limbs, which exerts a protective effect for distant organs [11–13]. Many clinical studies have suggested that RIC may have neuroprotection role in reducing stroke recurrence in patients with sICAS [14–16]. The European Stroke Organisation guidelines for intracranial atherosclerotic disease (ICAD) recommend ischemic conditioning as an adjuvant to best medical management [17]. Despite the weak strength of the recommendation and the need for further investigation, it shows that RIC is a promising treatment method for reducing the stroke recurrence in patients with sICAS. However, sex difference on the RIC treatment for sICAS patients is yet to be elucidated.

The RICA trial is the largest multicenter, randomized controlled trial for sICAS patients that found that daily RIC for 12 months significantly reduced the ischemic stroke recurrence in the per-protocol (PP) population [18]. Thus, in this predefined secondary analysis of the RICA trial, we sought to evaluate sex differences in ischemic stroke recurrence among 3,033 patients (1,079 women and 1,954 men) with sICAS. We also compared sex differences in the clinical efficacy of RIC among 1,409 patients (495 women and 914 men) with sICAS.

## Methods

### Study design and participants

The RICA trial was an multicenter, randomized clinical trial at 84 centers in China from 2015 October 28 to 2019 February 28. Previous publications have reported the design and methods of the RICA trial [18,19]. The main criteria for participation were that participants had to be aged between 40 and 80 years and had been diagnosed with transient ischemic attack (TIA) within 15 d before randomization or with ischemic stroke within 30 d before randomization. Stroke or TIA was due to 50% to 99% stenosis of a major intracranial artery. The key exclusion criteria were as follows: Vessels did not meet the inclusion criteria such as extracranial carotid artery stenosis ≥50% and cerebral venous thrombosis or stenosis, and contraindications to RIC [20]. The inclusion criteria and exclusion criteria are listed in Table S1. Before enrollment, informed consent must be signed.

### Procedures

Participants were randomly assigned on a 1:1 ratio to the RIC group (intervention group) or the sham RIC group (sham group). Both groups were administered once daily for 12 months after randomization. It was the patient’s discretion to use the study device after 12 months. Patients in the RIC group received the inflation pressure of 200 mmHg, and the sham RIC group received 60 mmHg. In addition to the study device, patients received other preventive treatments for stroke as determined appropriate by the investigators at their respective sites. Other preventive treatments were mainly in accordance with the Chinese guidelines for ischemic stroke and TIA from included antiplatelet therapy, blood pressure control, lipid regulation, and blood sugar control [21]. The complete study procedures have been reported previously. Patients attended clinical visits at 1, 3, 6, and 12 months and at the end of the study.

### Outcomes

The primary endpoint was the time to the initial ischemic stroke event following randomization. A new focal neurologic deficit of >24 h in duration, an exacerbation of an existing focal neurologic deficit of >24 h in duration, or a focal neurologic deficit of <24 h in duration with evidence of a new ischemic lesion based on neuroimaging was defined as ischemic stroke. Secondary endpoints included the composite secondary outcome, each component of the composite outcome and all-cause mortality.

### Statistical analysis

Sex differences in outcomes among participants with sICAS were analyzed in the intention-to-treat (ITT) population, encompassing all 3,033 individuals who were randomized. The effects of RIC and sham treatment on outcomes were separately analyzed in women and men within the PP population, consisting of 1,409 individuals, who adhered to at least 50% of the study intervention during the first year after randomization.

The baseline characteristics were reported, with continuous variables expressed as the mean and standard deviation (SD), and categorical variables shown as counts or percentage. The cumulative event rates were calculated using the Kaplan–Meier method and then evaluated in comparison with the results from stratified log-rank tests, based on the qualifying events (i.e., ischemic stroke or TIA). The Cox proportional hazards model with stratification was employed for calculating hazard ratios (HRs) and 95% confidence intervals (CIs). Differences in outcomes by sex were assessed using both unadjusted and adjusted stratified Cox models. The adjusted model included covariables that were consistent with the primary study, such as age, body mass index (BMI), and medical history.

The effect of RIC intervention versus sham intervention on outcomes was separately evaluated for women and men in unadjusted and adjusted stratified Cox models. The difference in the treatment effect between RIC and sham interventions for men and women was evaluated using the Cox model that included main effect terms (sex and intervention strategy) and an interaction term (sex and intervention strategy) for the outcome of interest. Statistical analyses were performed using R software (version 4.3.3). All tests were performed at a 2-sided 0.05 significance level.

## Results

### Baseline clinical characteristics

Of the 3,033 participants in the RICA trial, 1,079 (35.58%) were women and 1,954 (64.42%) were men. Table 1 shows the baseline clinical characteristics grouped by sex. Women were older (mean [SD] age 62.9 [8.8] years versus 60.0 [9.2] years) and had a higher BMI and higher prevalence of hypertension and diabetes compared to men. Lipid levels were also higher among female patients. Conversely, women were less likely to be current smokers. There were no significant differences in neurological scores [ABCD scale^2^ for TIA, modified Rankin scale (mRS) for ischemic stroke] and other comorbidities between the sexes.

**Table 1.** Baseline characteristics stratified by sex in the intention-to-treat population. Data are *n* (%), mean (SD), or median (interquartile range).

	Women (*n* = 1,079)	Men (*n* = 1,954)	*P* value
Age, years	62.9 (8.8)	60.0 (9.2)	<0.0001
Body mass index/(kg·m^-2^)	25.2 (3.4)	24.9 (2.9)	0.038
Qualifying event, *n*/%			
TIA	212 (19.6%)	375 (19.2%)	0.797
Ischemic stroke	867 (80.4%)	1,579 (80.8%)	··
Time from qualifying event to randomization, days			
TIA	9 (6.8–12.0)	9 (6.0–11.0)	0.456
Ischemic stroke	12 (8.0–17.0)	12 (8.0–16.0)	0.695
Neurological score			
ABCD scale^2^ for TIA ^a^*	4 (4–5)	4 (4–5)	0.321
mRS for ischemic stroke ^b^	1 (1–2)	1 (1–2)	0.076
Comorbidities (medical history and risk factors), *n*/%			
Previous ischemic stroke	267 (24.7%)	462 (23.6%)	0.525
Previous TIA	111 (10.3%)	225 (11.5%)	0.332
Previous myocardial infarction	88 (8.2%)	168 (8.6%)	0.726
Hypertension	932 (86.4%)	1,590 (81.4%)	0.001
Hyperlipidemia	717 (66.5%)	1,313 (67.2%)	0.706
Diabetes mellitus	418 (38.7%)	680 (34.8%)	0.034
Current or previous smoking	415 (38.5%)	1,478 (75.6%)	<0.0001
Symptomatic qualifying artery, *n* (%)			
Internal carotid	136 (12.6%)	281 (14.4%)	0.015
Middle cerebral	562 (52.1%)	980 (50.2%)	··
Basilar	218 (20.2%)	329 (16.8%)	··
Vertebral	118 (10.9%)	278 (14.2%)	··
Multiple arteries ^c^	45 (4.2%)	86 (4.4%)	··
Stenosis of qualifying artery ≥70%, *n*/% ^d^	423 (39.2%)	779 (39.9%)	0.750
Fasting blood glucose/mM	7.1 (3.0)	6.7 (2.7)	0.002
Blood pressure			
Systolic/mmHg	142.1 (16.3)	141.6 (16.7)	0.408
Diastolic/mmHg	84.3 (11.2)	85.4 (11.3)	0.011
Lipids			
LDL cholesterol/(mg·dl^-1^)	116.9 (34.6)	111.4 (31.4)	<0.0001
HDL cholesterol/(mg·dl^-1^)	46.3 (11.2)	42.2 (11.0)	<0.0001
Total cholesterol/(mg·dl^-1^)	191.6 (39.8)	179.7 (36.7)	<0.0001

TIA, transient ischemic attack; mRS, modified Rankin scale; LDL, low-density lipoprotein; HDL, high-density lipoprotein

^a^ Scores on the ABCD^2^ scale range from 0 to 7, with higher scores indicating a greater risk of stroke.

^b^ Scores on modified Rankin scale (mRS) range from 0 to 6, with higher scores indicating a greater stroke severity.

^c^ The affected arteries were a combination of the internal carotid and middle cerebral arteries, the vertebral and basilar arteries, or the left and right vertebral arteries.

^d^ Stenosis was quantified on the basis of a reading of the angiogram by the site investigators.

The baseline clinical characteristics according to sex and randomized treatment assignment in the PP population are reported in Table 2. This population included 1,409 participants, of which 914 (65.00%) were men and 495 (35.00%) were women. Among women, a higher prevalence of diabetes was found among those randomized to the RIC group compared to the sham group.

**Table 2.** Baseline characteristics by randomized treatment and sex in the per-protocol population. Data are *n* (%), mean (SD), or median (interquartile range).

	Women (*n* = 495)			Men (*n* = 914)		
	RIC	Control	*P* value	RIC	Control	*P* value
Age, years	63 (8.4)	62.5 (8.6)	0.508	60.7 (8.9)	60.4 (9.4)	0.674
Body mass index/(kg·m^-2^)	25.4 (3.3)	25.0 (3.2)	0.243	25.0 (2.6)	25.0 (3.0)	0.786
Qualifying event, *n*/%						
TIA	47 (19.7%)	56 (21.9%)	0.621	105 (22.6%)	100 (22.2%)	0.946
Ischemic stroke	192 (80.3%)	200 (78.1%)	··	359 (77.4%)	350 (77.8%)	··
Time from qualifying event to randomization, days						
TIA	11 (7.5–12)	8 (7–11.2)	0.830	9 (6–11)	8 (5.8–12)	0.229
Ischemic stroke	13 (9–18)	12 (8–17)	0.226	12 (8–16)	12 (8–16)	0.806
Neurological score						
ABCD scale^2^ for TIA ^a^	4 (4–5)	4 (4–5)	0.814	4 (4–5)	4 (4–5)	0.353
mRS for ischemic stroke ^b^	1 (1–2)	1 (1–2)	0.037	1 (1–2)	1 (1–2)	0.946
Comorbidities (medical history and risk factors), *n*/%						
Previous ischemic stroke	54 (22.6%)	65 (25.4%)	0.534	110 (23.7%)	110 (24.4%)	0.855
Previous TIA	27 (11.3%)	29 (11.3%)	1.000	61 (13.1%)	49 (10.9%)	0.344
Previous myocardial infarction	15 (6.3%)	20 (7.8%)	0.623	44 (9.5%)	43 (9.6%)	1.000
Hypertension	212 (88.7%)	222 (86.7%)	0.593	370 (79.7%)	371 (82.4%)	0.338
Hyperlipidemia	150 (62.8%)	173 (67.6%)	0.303	312 (67.2%)	303 (67.3%)	1.000
Diabetes mellitus	105 (43.9%)	89 (34.8%)	0.046	163 (35.1%)	164 (36.4%)	0.730
Current or previous smoking	91 (38.1%)	104 (40.6%)	0.625	348 (75.0%)	329 (73.1%)	0.565
Symptomatic qualifying artery, *n*/%						
Internal carotid	24 (10.0%)	30 (11.7%)	0.867	71 (15.3%)	68 (15.1%)	0.731
Middle cerebral	125 (52.3%)	133 (52.0%)	··	230 (49.6%)	217 (48.2%)	··
Basilar	50 (20.9%)	47 (18.4%)	··	71 (15.3%)	82 (18.2%)	··
Vertebral	27 (11.3%)	34 (13.3%)	··	72 (15.5%)	61 (13.6%)	··
Multiple arteries ^c^	13 (5.4%)	12 (4.7%)	··	20 (4.3%)	22 (4.9%)	··
Stenosis of qualifying artery ≥70%, *n*/% ^d^	90 (37.7%)	105 (41%)	0.501	183 (39.4%)	176 (39.1%)	0.973
Fasting blood glucose/mM	7.3 (3.1)	6.9 (3.0)	0.171	6.7 (2.6)	6.7 (2.9)	0.930
Blood pressure						
Systolic/mmHg	141.3 (16.9)	142.1 (16.5)	0.594	142.5 (16.2)	141.2 (16.7)	0.241
Diastolic/mmHg	83.3 (11.5)	84.0 (10.9)	0.434	85.8 (11.2)	84.7 (10.6)	0.120
Lipids						
LDL cholesterol/(mg·dl^-1^)	116.9 (33.8)	116.9 (33.8)	0.996	111.3 (33.2)	112.7 (33.2)	0.540
HDL cholesterol/(mg·dl^-1^)	45.8 (11.2)	46.0 (11.8)	0.853	41.9 (11.1)	42.8 (11.6)	0.225
Total cholesterol/(mg·dl^-1^)	191.5 (38.1)	191.9 (38.7)	0.919	180.3 (38.1)	181.0 (39.4)	0.784

^a^ Scores on the ABCD^2^ scale range from 0 to 7, with higher scores indicating a greater risk of stroke.

^b^ Scores on modified Rankin scale (mRS) range from 0 to 6, with higher scores indicating a greater stroke severity.

^c^ The affected arteries were a combination of the internal carotid and middle cerebral arteries, the vertebral and basilar arteries, or the left and right vertebral arteries.

^d^ Stenosis was quantified on the basis of a reading of the angiogram by the site investigators.

### Outcomes by sex in the ITT population

**Fig. 1.**
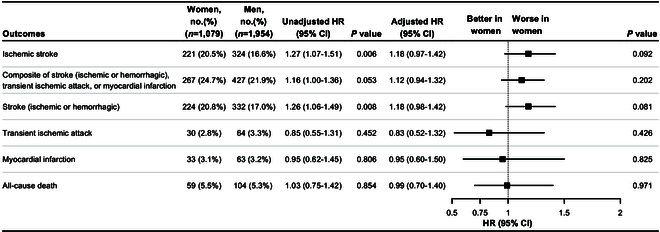
Association of sex with clinical outcomes in the intention-to-treat population. *Adjusted by age, body mass index, time from qualifying event to randomization, previous ischemic stroke, previous transient ischemic attack, previous myocardial infarction, systolic blood pressure, low-density lipoprotein cholesterol, fasting blood glucose, smoking status, symptomatic qualifying artery, and stenosis degree of qualifying artery.

During the mean 3.5-year follow-up period, women had a higher unadjusted ischemic stroke recurrence rate than men (20.5% versus 16.6%; unadjusted HR, 1.27; [95% CI, 1.07 to 1.51]; *P* = 0.006) and stroke (ischemic or hemorrhagic) occurs more frequently in women than in men (20.8% versus 17.0%; unadjusted HR, 1.26; [95% CI, 1.06 to 1.49]; *P* = 0.008). However, after adjusting for baseline confounders, ischemic stroke (adjusted HR, 1.18; [95% CI, 0.97 to 1.42]; *P* = 0.092) and stroke (ischemic or hemorrhagic) (adjusted HR, 1.18; [95% CI, 0.98 to 1.42; *P* = 0.081]) recurrence did not differ between the sexes. Additionally, no statistically significant sex differences were found for the other secondary endpoints (Fig. 1).

### Outcomes by sex and randomized treatment assignment in the PP population

**Fig. 2.**
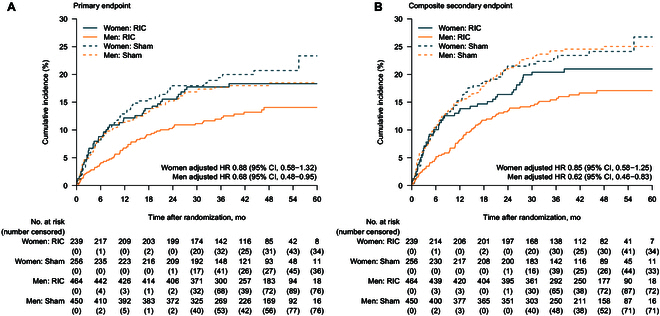
Kaplan–Meier event curve for the primary endpoint and the composite secondary endpoint by sex in the per-protocol population. (A) Primary endpoint (ischemic stroke). (B) Composite secondary endpoint (stroke, transient ischemic attack, or myocardial infarction). RIC, remote ischemic conditioning; HR, hazard ratio.

The clinical outcomes for male and female patients in the PP population are shown in Table 3. The RIC intervention was associated with a lower rate of ischemic stroke recurrence in men (unadjusted HR, 0.70; [95% CI, 0.50 to 0.97]; *P* = 0.034; adjusted HR, 0.68; [95% CI, 0.48 to 0.95]; *P* = 0.025) compared to the sham group, but this effect was not seen in women (unadjusted HR, 0.89; [95% CI, 0.59 to 1.33]; *P* = 0.558; adjusted HR, 0.88; [95% CI, 0.58 to 1.32]; *P* = 0.539), with no significant interaction between treatment allocation and sex (*P* for interaction = 0.379) at a mean follow-up of 3.5 years after randomization (Table 3 and Fig. 2).

**Table 3.** Outcomes by sex and randomized treatment assignment in the per-protocol population. Data are number of first events (%).

Outcomes	RIC	Control	HR (95% CI)	*P* value	Adjusted HR ^a^ (95% CI)	Adjusted *P* value	*P* value for interaction
Ischemic stroke							0.379
Women	43 (18.0%)	52 (20.3%)	0.89 (0.59–1.33)	0.558	0.88 (0.58–1.32)	0.539	
Men	60 (12.9%)	80 (17.8%)	0.70 (0.50–0.97)	0.034	0.68 (0.48–0.95)	0.025	
Composite of stroke (ischemic or hemorrhagic), TIA, or myocardial infarction							0.211
Women	49 (20.5%)	61 (23.8%)	0.86 (0.59–1.25)	0.418	0.85 (0.58–1.25)	0.410	
Men	75 (16.2%)	109 (24.2%)	0.63 (0.47–0.84)	0.002	0.62 (0.46–0.83)	0.001	
Stroke (ischemic or hemorrhagic)							0.314
Women	44 (18.4%)	52 (20.3%)	0.91 (0.61–1.36)	0.634	0.90 (0.60–1.35)	0.605	
Men	61 (13.1%)	82 (18.2%)	0.69 (0.50–0.96)	0.028	0.67 (0.48–0.94)	0.020	
TIA							0.871
Women	4 (1.7%)	6 (2.3%)	0.71 (0.20–2.51)	0.592	0.62 (0.16–2.32)	0.477	
Men	10 (2.2%)	15 (3.3%)	0.64 (0.29–1.42)	0.266	0.60 (0.27–1.34)	0.209	
Myocardial infarction							0.620
Women	6 (2.5%)	7 (2.7%)	0.93 (0.31–2.77)	0.896	1.09 (0.34–3.49)	0.887	
Men	11 (2.4%)	16 (3.6%)	0.66 (0.31–1.42)	0.281	0.70 (0.32–1.52)	0.368	
All-cause death							0.439
Women	14 (5.9%)	13 (5.1%)	1.16 (0.54–2.46)	0.704	1.09 (0.51–2.37)	0.819	
Men	19 (4.1%)	23 (5.1%)	0.79 (0.43–1.45)	0.447	0.79 (0.43–1.46)	0.448	

RIC, remote ischemic conditioning; HR, hazard ratio; CI, confidence internal

^a^ Adjusting for confounders included the baseline variables age, body mass index, time from qualifying event to randomization, previous ischemic stroke, previous transient ischemic attack, previous myocardial infarction, systolic blood pressure, low-density lipoprotein cholesterol, fasting blood glucose, smoking status, symptomatic qualifying artery, and stenosis degree of qualifying artery.

The composite secondary outcome events were numerically lower with the RIC intervention in women (49 [20.5%] versus 61 [23.8%]; unadjusted HR, 0.86; [95% CI, 0.59 to 1.25]; *P* = 0.418; adjusted HR, 0.85; [95% CI, 0.58 to 1.25]; *P* = 0.410) and significantly lower in men (75 [16.2%] versus 109 [24.2%]; unadjusted HR, 0.63; [95% CI, 0.47 to 0.84]; *P* = 0.002; adjusted HR, 0.62; [95% CI, 0.46 to 0.83]; *P* = 0.001), with no significant interaction (*P* for interaction = 0.211) (Fig. 2). No evidence of heterogeneity in treatment effects by sex was found for other secondary endpoints except for stroke (ischemic or hemorrhagic) (*P* = 0.314 for interaction), in which the RIC group demonstrated a lower risk of stroke recurrence in men (unadjusted HR, 0.69; [95% CI, 0.50 to 0.96]; *P* = 0.028; adjusted HR, 0.67; [95% CI, 0.48 to 0.94]; *P* = 0.020) but not in women (unadjusted HR, 0.91; [95% CI, 0.61 to 1.36]; *P* = 0.634; adjusted HR, 0.90; [95% CI, 0.60 to 1.35]; *P* = 0.605) (Fig. S1).

### Outcomes in age-stratified and degree of stenosis stratified in the PP population

To further evaluate sex-related differences in ischemic stroke recurrence during the 3.5-year follow-up period, we stratified participants by age and degree of stenosis. Among patients aged 65 years and above, we found no significant differences in primary and secondary outcomes between male and female patients (Table S2). However, in men aged younger than 65 years old, RIC had reduced the stroke (34 RIC [10.8%] versus 47 sham [16.0%]; adjusted HR, 0.62; [95% CI, 0.39 to 0.97]; *P* = 0.034; *P* for interaction = 0.966) and composite secondary outcome event (41 RIC [13.0%] versus 63 sham [21.4%]; adjusted HR, 0.53; [95% CI, 0.36 to 0.79]; *P* = 0.002; *P* for interaction = 0.488) recurrence rate than the sham group (Table S3).

Among patients with the degree of stenosis of less than 70%, the primary and secondary endpoints did not differ between the RIC and sham groups (Table S4). Clinical outcomes in patients with a degree of stenosis of 70% or more are shown in Table S5. The risk of ischemic stroke recurrence (adjusted HR, 0.58; [95% CI, 0.36 to 0.93]; *P* = 0.023; *P* for interaction = 0.976), composite secondary outcome events (adjusted HR, 0.55; [95% CI, 0.37 to 0.84]; *P* = 0.006; *P* for interaction = 0.488), and stroke (adjusted HR, 0.57; [95% CI, 0.36 to 0.91]; *P* = 0.017; *P* for interaction = 0.966) was significantly reduced in men but not in women.

## Discussion

This prespecified secondary analysis, which included 3,033 patients with sICAS from 84 centers in China, revealed several key findings. First, notable differences in baseline characteristics were observed between sexes, with women being older and having a more frequent occurrence of stroke risk factors in comparison to men. After adjusting for these baseline characteristics, no significant difference in the risk of stroke recurrence was found between men and women patients with sICAS over the 3.5-year follow-up period. Second, subgroup analyses in the PP population, RIC reduced the risk of stroke recurrence among male patients. However, this trend was not observed in female patients.

Regardless of randomization strategy, our findings were consistent with those of the CICAS (Chinese Intracranial Atherosclerosis Study) [22] and SAMMPRIS trial [23], both of which also reported no sex-based differences in outcomes for patients with sICAS. Conversely, a subgroup analysis of the WASID trial [24] revealed an elevated risk of ischemic stroke recurrence among women with sICAS, even after adjusting for various factors, such as social context (marital status), lifestyle, vascular risks, angiographic findings, and qualifying event features. One possible explanation for the differing results is that our study did not adjust for variables related to social context. An investigation into the disparities in stroke outcomes based on sex and the associated factors among individuals suffering from acute ischemic stroke highlights the importance of social context, particularly for women who have experienced a stroke [25,26]. Women often have worse outcomes after stroke, which may be due to factors such as living alone, being unmarried, widowed, and having a low socioeconomic status [24]. Furthermore, an unfavorable social context may result in inadequate treatment for women [25]. Another explanation is that our study was conducted from 2015 to 2019, whereas the WASID trial was conducted from 1999 to 2003. Research suggests that sex differences in outcomes of patients with stroke have narrowed significantly over the past decade, particularly between 2015 and 2018 [25]. This could also be another significant reason why we found no significant sex differences in ischemic stroke recurrence among patients with sICAS in our study.

The RICAMIS trial [27], which controlled for various factors, assessed the effect of sex differences on the efficacy of RIC treatment. The findings indicated that female patients receiving RIC could potentially have a higher probability of achieving superior functional results at 90 d compared to participants in the control group. However, significant interaction effect between sex and treatment strategy was not observed. This outcome of RICAMIS study may appear to be at odds with the RICA trial. The RICA trial has not found interaction between treatment strategy and sex, and suggests that RIC is beneficial for men. The difference pertains to the study population (RICA trial: sICAS versus RICAMIS trial: acute ischemic stroke), modes of RIC intervention (RICA trial: once daily for 12 months versus RICAMIS trial: 10 to 14 d), and protocol adherence [18,27].

Of note in the RICA trial, the rate of ischemic stroke recurrence was lower in men randomized to the RIC group versus the sham RIC group with no significant interaction between sex and treatment strategy with the following explanations. First, there seemed to be a trend suggesting a difference between the RIC and sham group among women, yet statistical significance was not achieved, possibly due to the underrepresentation of female participants, who constituted only 35% of the study sample. Second, RICAMIS subgroup analyses suggested that RIC effectiveness decreases with age [28], and in our study, the women were older. This finding suggests that the older age of the enrolled women in the RICA trial may have contributed to this outcome. Lastly, animal studies have indicated that RIC may have better neuroprotective effects in male animals [29]; however, the specific mechanisms have not yet been explored. A basic research study on cardiac ischemia/reperfusion and ischemic post-conditioning has identified that superoxide production and apoptosis processes are important factors to the sex-related differences [30]. Future research is required to explore this pathway in ICAS.

This study has some limitations. First, the randomization process was not stratified by sex, and the underrepresentation of female participants could potentially result in a type II error, indicating that results should be interpreted with caution. We will continue to focus on this aspect in future studies and take steps to ensure that the population of samples is gender-balanced. Second, significant differences in baseline risk factors, such as diabetes and BMI, were observed between sex and among treatment groups, even after baseline adjustments. These unaccounted confounding factors, particularly social context, may have influenced the results. Third, as these secondary analyses were prespecified, our results should be considered preliminary and serve as a basis for generating the hypotheses. Consequently, the observed differences in RIC’s impact on stroke recurrence between sexes may be incidental and require further investigation. Lastly, since the study focused on a Chinese cohort, its applicability to other populations may be limited, highlighting the necessity for further research to confirm these results in other populations.

## Conclusion

In this prespecified secondary analysis of the RICA trial, no significant sex differences were found in the ischemic stroke recurrence during a mean follow-up of 3.5 years. Men with good adherence to RIC may reduce the recurrence of ischemic stroke, while women did not show any significant benefits. This finding should be considered exploratory, and further validation through randomized controlled trials is necessary to determine whether RIC can effectively reduce the recurrence rate of ischemic stroke in women with sICAS.

## Data Availability

The individual deidentified participant data that support the findings of this study will be made accessible to any qualified external researcher upon request, following approval of a proposal by the steering committee. Proposals for data access should be submitted to rica_group@ccmu.edu.cn.
