# Supplementary material for: Sex-Related Difference in Outcomes of Remote Ischemic Conditioning for Symptomatic Intracranial Atherosclerotic Stenosis
Source: Cyborg Bionic Syst. 2025 Jun 6;6:0275. doi: 10.34133/cbsystems.0275 (PMC12141781; doi:10.34133/cbsystems.0275)
Supplement: Supplementary 1 — Tables S1 to S5 Fig. S1 [file cbsystems.0275.f1.zip › Supplemental Figure 1.pdf]

A. Stroke (ischemic or hemorrhagic)

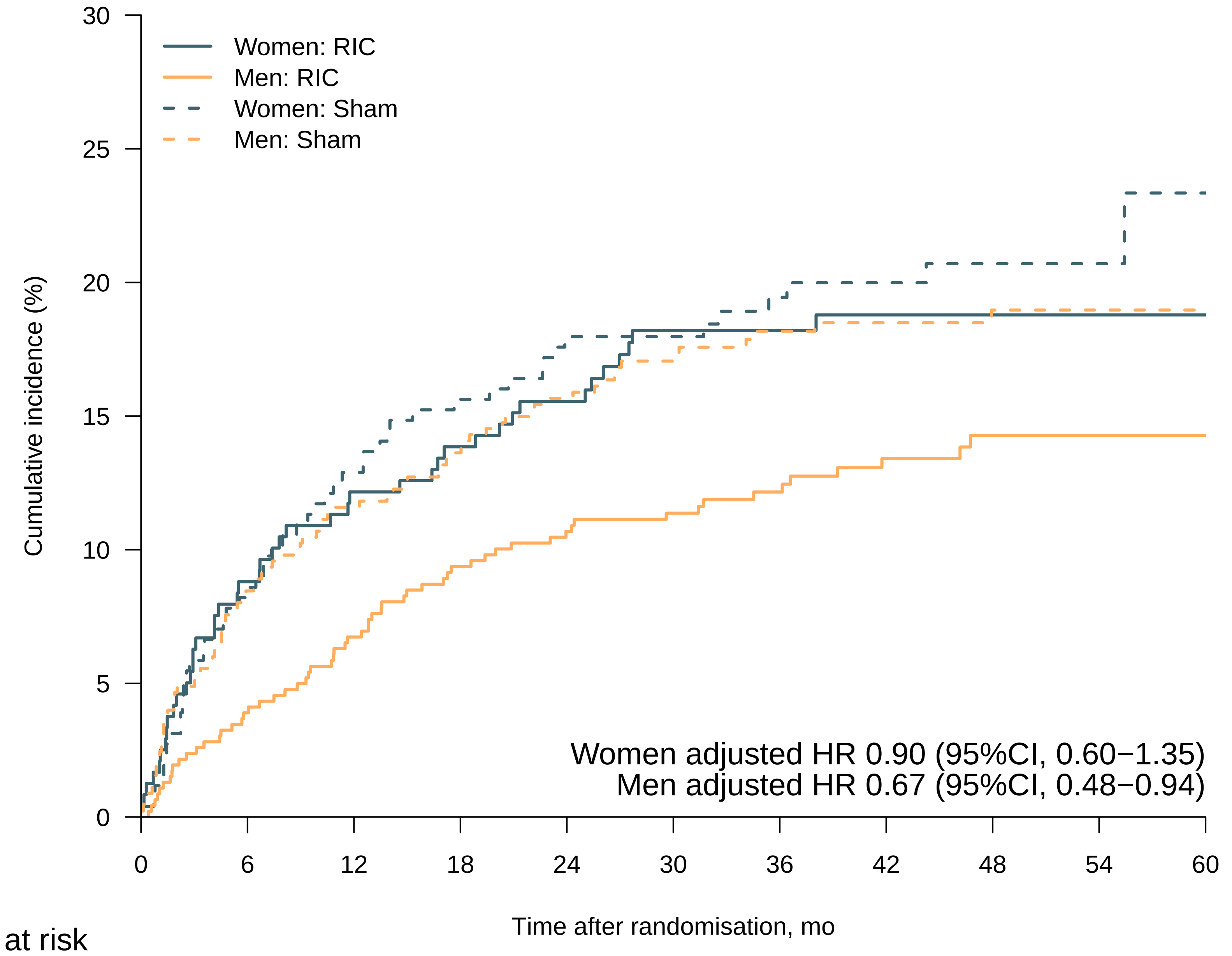

No. at risk<br>mber censored)											
Women: RIC	239	217	209	203	199	173	142	116	85	42	8
	(0)	(1)	(0)	(2)	(0)	(20)	(31)	(25)	(31)	(43)	(34)
Women: Sham	256	235	223	216	209	192	148	121	93	48	11
	(0)	(0)	(0)	(0)	(1)	(17)	(41)	(26)	(27)	(45)	(36)
Men: RIC	464	442	426	413	405	370	299	256	182	93	18
	(0)	(4)	(3)	(1)	(2)	(32)	(68)	(39)	(72)	(89)	(75)
Men: Sham	450	410	391	382	371	325	268	226	169	92	16
	(0)	(2)	(5)	(0)	(2)	(40)	(53)	(41)	(56)	(77)	(76)

B. Transient ischemic attack

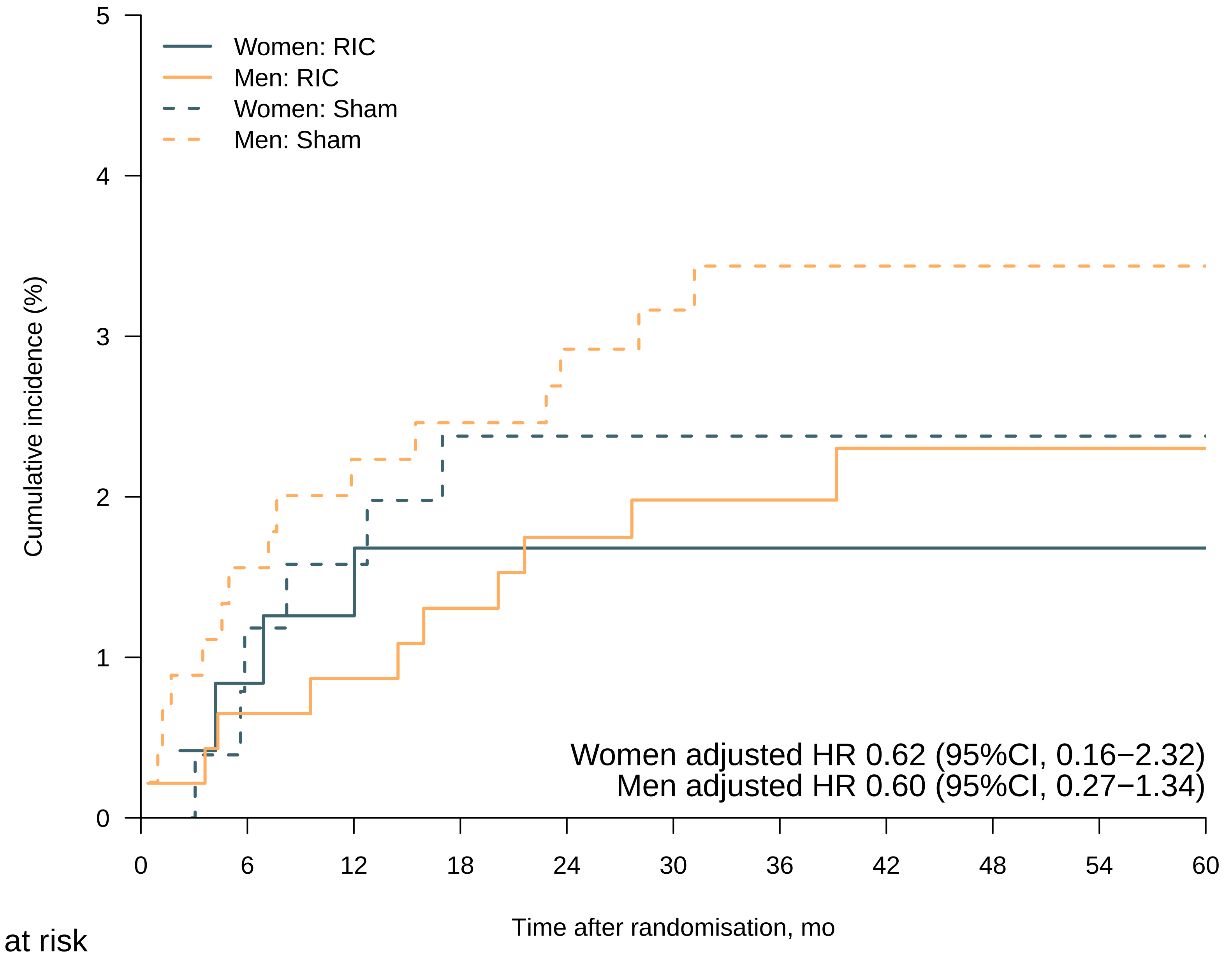

No. at risk<br>(number censored)											
Women: RIC	239	236	234	230	228	204	164	138	100	52	9
	(0)	(1)	(1)	(3)	(2)	(24)	(40)	(26)	(38)	(48)	(43)
Women: Sham	256	250	247	244	243	224	173	142	111	57	14
	(0)	(3)	(2)	(1)	(1)	(19)	(51)	(31)	(31)	(54)	(43)
Men: RIC	464	457	453	450	444	410	330	286	207	107	24
	(0)	(4)	(3)	(1)	(4)	(33)	(80)	(43)	(79)	(100)	(83)
Men: Sham	450	440	431	427	423	375	313	265	200	106	19
	(0)	(3)	(6)	(3)	(2)	(47)	(61)	(48)	(65)	(94)	(87)

C. Myocardial infarction

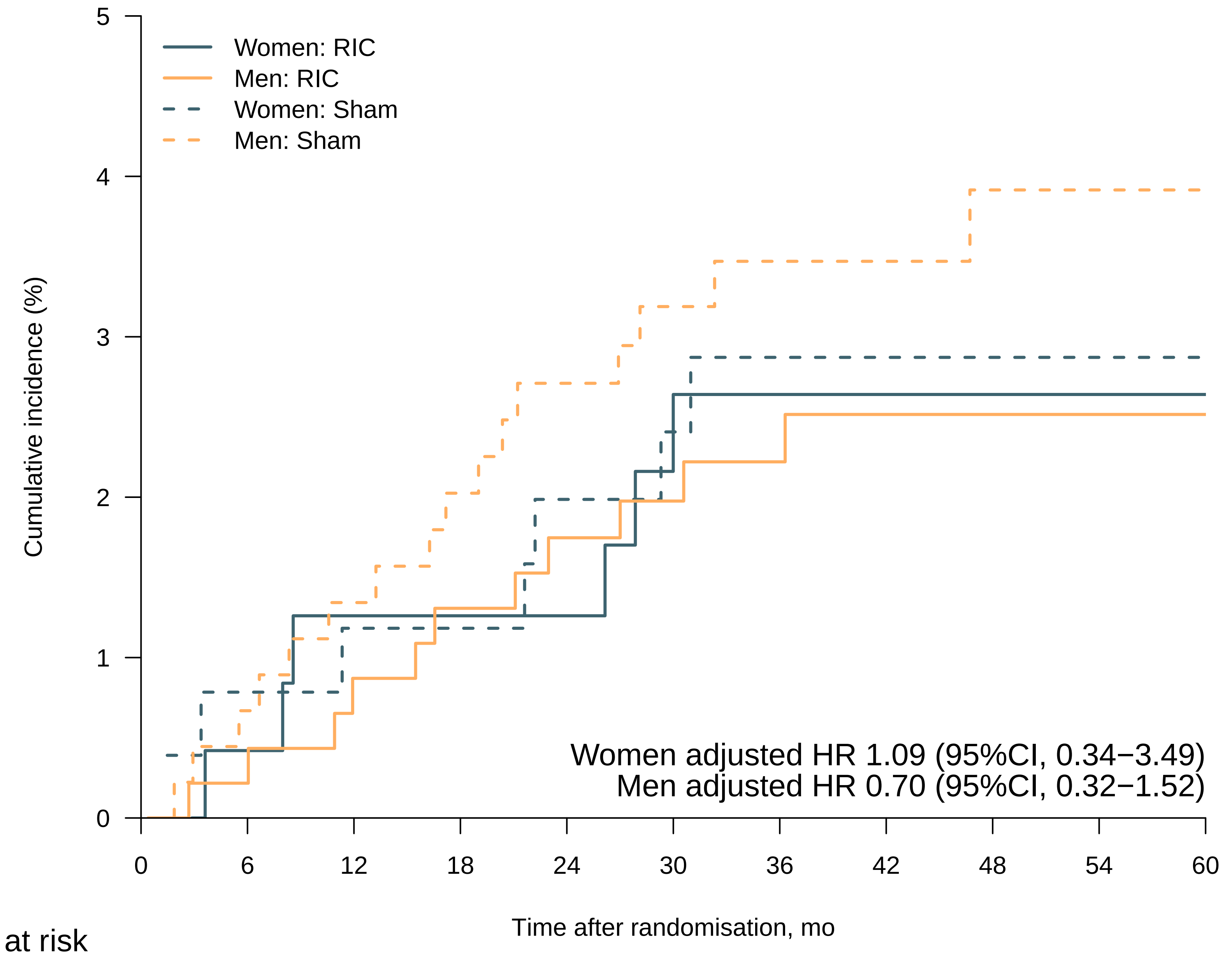

No. at risk<br>mber censored)											
Women: RIC	239	237	234	231	229	203	162	136	99	51	8
	(0)	(1)	(1)	(3)	(2)	(23)	(41)	(26)	(37)	(48)	(43)
Women: Sham	256	251	248	247	243	224	172	142	112	59	14
	(0)	(3)	(2)	(1)	(2)	(18)	(51)	(30)	(30)	(53)	(45)
Men: RIC	464	460	454	452	447	411	334	291	210	111	24
	(0)	(3)	(3)	(0)	(3)	(35)	(76)	(42)	(81)	(99)	(87)
Men: Sham	450	444	435	429	426	377	314	265	202	107	19
	(0)	(3)	(6)	(3)	(0)	(47)	(62)	(49)	(62)	(95)	(88)

D. All−cause death

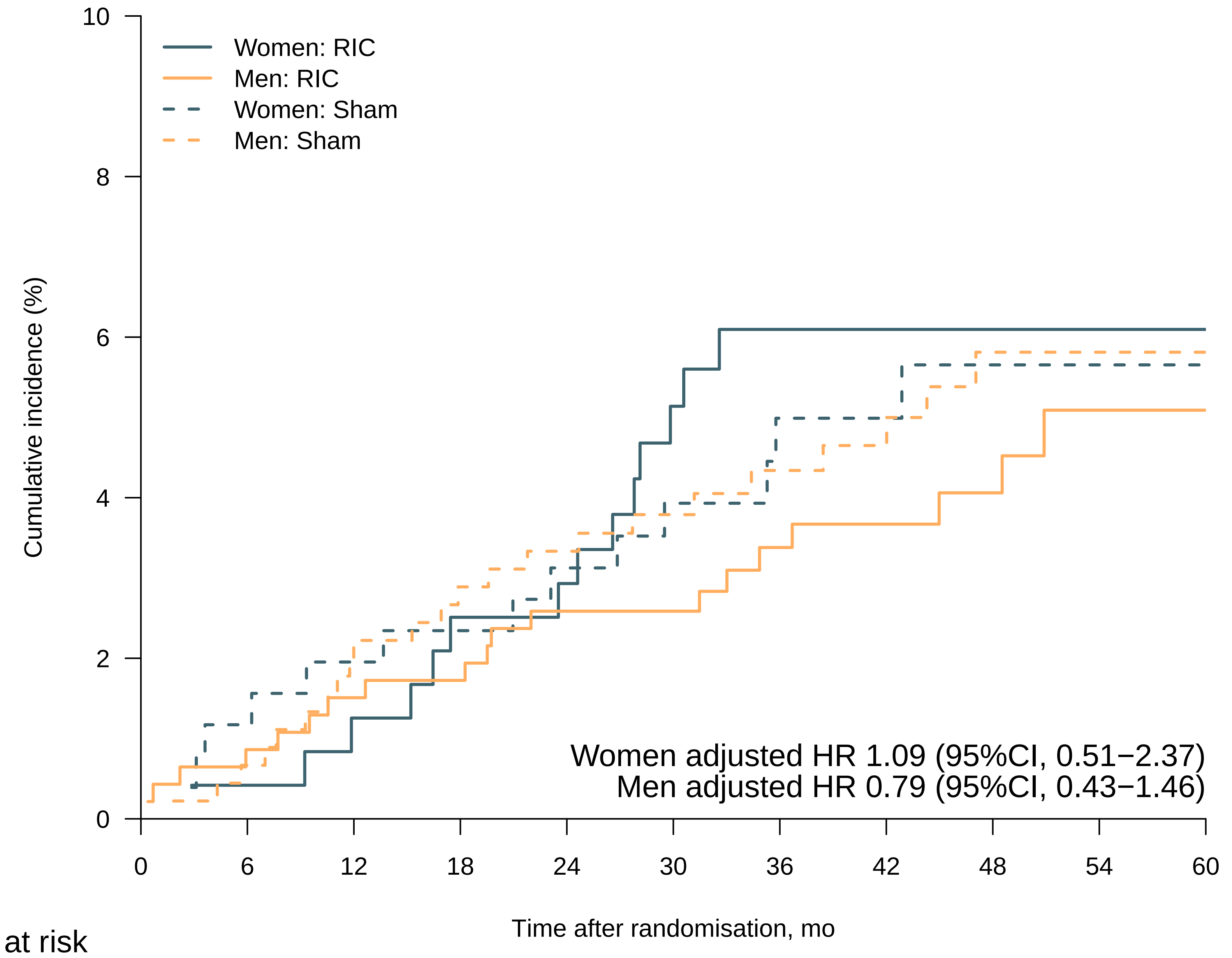

No. at risk<br>(number censored)											
Women: RIC	239	238	236	233	231	207	165	139	101	52	9
	(0)	(0)	(0)	(0)	(1)	(19)	(40)	(26)	(38)	(49)	(43)
Women: Sham	256	253	251	250	248	229	177	146	115	60	14
	(0)	(0)	(0)	(0)	(0)	(17)	(50)	(31)	(30)	(55)	(46)
Men: RIC	464	460	457	456	452	417	337	294	213	112	24
	(0)	(0)	(0)	(0)	(0)	(35)	(77)	(42)	(80)	(99)	(88)
Men: Sham	450	447	440	437	435	388	324	273	207	109	19
	(0)	(0)	(0)	(0)	(0)	(45)	(62)	(50)	(63)	(98)	(90)
