# Supplementary material for: Sex-Related Difference in Outcomes of Remote Ischemic Conditioning for Symptomatic Intracranial Atherosclerotic Stenosis
Source: Cyborg Bionic Syst. 2025 Jun 6;6:0275. doi: 10.34133/cbsystems.0275 (PMC12141781; doi:10.34133/cbsystems.0275)
Supplement: Supplementary 1 — Tables S1 to S5 Fig. S1 [file cbsystems.0275.f1.zip › SUPPLEMENTARY APPENDIX-CBS.docx]

**SUPPLEMENTARY APPENDIX**

**Sex-Related Difference in Outcomes of Remote Ischemic Conditioning for Symptomatic Intracranial Atherosclerotic Stenosis**

**Table of Contents**

[Supplemental Table 1. Inclusion and Exclusion Criteria 2](#_Toc12031)

[Supplemental Table 2. Clinical outcomes after randomization in male and female patients with age ≥ 65 in the per-protocol population 4](#_Toc8964)

[Supplemental Table 3. Clinical outcomes after randomization in male and female patients with age < 65 in the per-protocol population 5](#_Toc23017)

[Supplemental Table 4. Clinical outcomes after randomization in male and female patients without degree of stenosis < 70% in the per-protocol population 6](#_Toc22223)

[Supplemental Table 5. Clinical outcomes after randomization in male and female patients with degree of stenosis ≥ 70% in the per-protocol population 7](#_Toc11278)

[Supplemental Figure 1. Kaplan-Meier event curve for the other secondary endpoint by sex in the per-protocol population 8](#_Toc30313)

**Supplemental Table 1. Inclusion and** **Exclusion** **Criteria**

| **Inclusion Criteria** |
| --- |
| Men and women between 40 and 80 years of age. |
| Patients who suffered an ischemic stroke or TIA prior to enrollment. |
| Patients who suffered an ischemic stroke within 30 days prior to enrollment with a baseline mRS score ≤4. |
| Patients who suffered a TIA within 15 days prior to enrollment with a baseline ABCD^2^ score ≥4. |
| Qualifying event attributable to symptomatic intracranial atherosclerotic stenosis (50%-99%) of carotid artery, middle cerebral artery (M1 segment), vertebral artery, or basilar artery that has been documented by magnetic resonance angiography or computed tomography angiography. |
| Informed consent obtained. |
| **Exclusion Criteria** |
| Thrombolytic therapy within 24 hours prior to enrollment. |
| Progressive neurological signs within 24 hours prior to enrollment. |
| Cerebral venous thrombosis/stenosis. |
| Intracranial arterial stenosis due to arterial dissection; Moyamoya disease; any known vasculitic disease; herpes zoster, varicella zoster, or other viral vasculopathy; neurosyphilis; any other intracranial infection; intracranial stenosis associated with cerebral spinal fluid pleocytosis; radiation induced vasculopathy; fibromuscular dysplasia; sickle cell disease; neurofibromatosis; benign angiopathy of central nervous system; postpartum angiopathy; suspected vasospastic process; or suspected recanalized embolus. |
| Any of the following unequivocal cardiac source of embolism: rheumatic mitral disease with or without aortic stenosis, prosthetic heart valves, atrial fibrillation, atrial flutter, sick sinus syndrome, left atrial myxoma, patent foramen ovale, left ventricular mural thrombus or valvular vegetation, congestive heart failure, bacterial endocarditis, or any other severe cardiovascular condition. |
| Uncontrolled severe hypertension, defined by sitting systolic blood pressure >180mmHg and/or sitting diastolic blood pressure >110mmHg after medication. |
| Any of the following unequivocal cardiac source of embolism: rheumatic mitral disease with or without aortic stenosis, prosthetic heart valves, atrial fibrillation, atrial flutter, sick sinus syndrome, left atrial myxoma, patent foramen ovale, left ventricular mural thrombus or valvular vegetation, congestive heart failure, bacterial endocarditis, or any other severe cardiovascular condition. |
| Uncontrolled severe hypertension, defined by sitting systolic blood pressure >180mmHg and/or sitting diastolic blood pressure >110mmHg after medication. |
| Intracranial neoplasm, cerebral aneurysm, or arteriovenous malformation. |
| Retinal hemorrhage or visceral bleeding within 30 days prior to enrollment. |
| Severe hemostatic disorder or severe coagulation dysfunction. |
| Subclavian arterial stenosis ≥50% or subclavian steal syndrome. |
| Extracranial stenosis ≥50%. |
| Treatment of a target lesion with a stent, angioplasty, or other mechanical device prior to enrollment or intent to perform such a procedure within 12 months after enrollment. |
| Major surgery, including cardiac and open femoral, aortic, or carotid surgery, within 30 days prior to enrollment or intent to undergo within 12 months after enrollment. |
| Contraindication for remote ischemic conditioning, including severe soft tissue injury, fracture, or peripheral vascular disease in the upper limbs. |
| Life expectancy <3 years. |
| Women who were pregnant or breast-feeding at the time of enrollment or anytime during the study period. |
| Unwilling to comply with the treatment or follow-up assessments. |
| Participating in another clinical trial within 3 months prior to enrollment of this clinical trial. |
| Any patient deemed unsuitable for enrollment by the investigators. |

**Supplemental Table 2. Clinical outcomes after randomization in male and female patients with age ≥ 65 in the per-protocol population**

|  | **Women** | | | | | |  | **Men** | | | | | |  |
| --- | --- | --- | --- | --- | --- | --- | --- | --- | --- | --- | --- | --- | --- | --- |
|  | **RIC (n = 102)** | **Control (n = 109)** | **Unadjusted HR (95% CI)** | **Unadjusted *p* value** | **Adjusted HR (95% CI)** | **Adjusted *p* value** |  | **RIC (n = 148)** | **Control (n = 156)** | **Unadjusted HR (95% CI)** | **Unadjusted *p* value** | **Adjusted HR (95% CI)** | **Adjusted *p* value** | ***p* for  interaction** |
| Ischemic stroke | 19 (18.6%) | 24 (22.0%) | 0.83 (0.45-1.52) | 0.546 | 0.77 (0.41-1.43) | 0.407 |  | 26 (17.6%) | 34 (21.8%) | 0.78 (0.47-1.31) | 0.347 | 0.80 (0.46-1.36) | 0.404 | 0.976 |
| Composite of stroke (ischemic or hemorrhagic),  TIA, or myocardial infarction | 20 (19.6%) | 27 (24.8%) | 0.76 (0.43-1.36) | 0.358 | 0.70 (0.38-1.27) | 0.238 |  | 34 (23.0%) | 46 (29.5%) | 0.74 (0.48-1.16) | 0.191 | 0.74 (0.46-1.17) | 0.198 | 0.488 |
| Stroke (ischemic or hemorrhagic) | 19 (18.6%) | 24 (22.0%) | 0.83 (0.45-1.52) | 0.546 | 0.77 (0.41-1.43) | 0.407 |  | 27 (18.2%) | 35 (22.4%) | 0.79 (0.48-1.31) | 0.356 | 0.80 (0.47-1.36) | 0.405 | 0.966 |
| TIA | 1 (1.0%) | 3 (2.8%) | 0.33 (0.03-3.13) | 0.306 | - | - |  | 5 (3.4%) | 6 (3.8%) | 0.89 (0.27-2.91) | 0.843 | 0.81 (0.23-2.88) | 0.744 | 0.097 |
| Myocardial infarction | 2 (2.0%) | 3 (2.8%) | 0.75 (0.12-4.54) | 0.756 | 0.24 (0.01-6.09) | 0.390 |  | 6 (4.1%) | 7 (4.5%) | 0.91 (0.31-2.71) | 0.864 | 0.95 (0.30-3.01) | 0.931 | 0.116 |
| All-cause death | 9 (8.8%) | 11 (10.1%) | 0.89 (0.37-2.14) | 0.789 | 0.94 (0.37-2.41) | 0.900 |  | 9 (6.1%) | 8 (5.1%) | 1.25 (0.48-3.24) | 0.646 | 1.29 (0.46-3.59) | 0.632 | 0.912 |

RIC, remote ischemic conditioning. HR, hazard ratio. CI, confidence internal. TIA, transient ischemic attack. The number of the outcome is too small to calculate HRs or adjusted HRs.

**Supplemental Table 3. Clinical outcomes after randomization in male and female patients with age < 65 in the per-protocol population**

|  | **Women** | | | | | |  | **Men** | | | | | |  |
| --- | --- | --- | --- | --- | --- | --- | --- | --- | --- | --- | --- | --- | --- | --- |
|  | **RIC (n = 137)** | **Control (n = 147)** | **Unadjusted HR (95% CI)** | **Unadjusted *p* value** | **Adjusted HR (95% CI)** | **Adjusted *p* value** |  | **RIC (n = 316)** | **Control (n = 294)** | **Unadjusted HR (95% CI)** | **Unadjusted *p* value** | **Adjusted HR (95% CI)** | **Adjusted *p* value** | ***p* for  interaction** |
| Ischemic stroke | 24 (17.5%) | 28 (19.0%) | 0.93 (0.54-1.60) | 0.793 | 0.93 (0.53-1.63) | 0.788 |  | 34 (10.8%) | 46 (15.6%) | 0.66 (0.42-1.03) | 0.063 | 0.63 (0.40-0.99) | 0.045 | 0.976 |
| Composite of stroke (ischemic or hemorrhagic),  TIA, or myocardial infarction | 29 (21.2%) | 34 (23.1%) | 0.93 (0.56-1.52) | 0.758 | 0.94 (0.56-1.57) | 0.804 |  | 41 (13.0%) | 63 (21.4%) | 0.57 (0.38-0.84) | 0.004 | 0.53 (0.36-0.79) | 0.002 | 0.488 |
| Stroke (ischemic or hemorrhagic) | 25 (18.2%) | 28 (19.0%) | 0.97 (0.57-1.66) | 0.910 | 0.96 (0.55-1.69) | 0.899 |  | 34 (10.8%) | 47 (16.0%) | 0.64 (0.41-1.00) | 0.049 | 0.62 (0.39-0.97) | 0.034 | 0.966 |
| TIA | 3 (2.2%) | 3 (2.0%) | 1.07 (0.22-5.32) | 0.931 | 0.29 (0.03-2.54) | 0.264 |  | 5 (1.6%) | 9 (3.1%) | 0.51 (0.17-1.51) | 0.214 | 0.47 (0.15-1.53) | 0.209 | 0.097 |
| Myocardial infarction | 4 (2.9%) | 4 (2.7%) | 1.10 (0.27-4.39) | 0.894 | 2.26 (0.45-11.35) | 0.323 |  | 5 (1.6%) | 9 (3.1%) | 0.51 (0.17-1.52) | 0.217 | 0.59 (0.19-1.86) | 0.370 | 0.116 |
| All-cause death | 5 (3.6%) | 2 (1.4%) | 2.73 (0.53-14.05) | 0.212 | 2.10 (0.27-16.03) | 0.476 |  | 10 (3.2%) | 15 (5.1%) | 0.61 (0.27-1.35) | 0.216 | 0.57 (0.24-1.33) | 0.192 | 0.912 |

RIC, remote ischemic conditioning. HR, hazard ratio. CI, confidence internal. TIA, transient ischemic attack.

**Supplemental Table 4. Clinical outcomes after randomization in male and female patients without degree of stenosis < 70% in the per-protocol population**

|  | **Women** | | | | | |  | **Men** | | | | | |  |
| --- | --- | --- | --- | --- | --- | --- | --- | --- | --- | --- | --- | --- | --- | --- |
|  | **RIC (n = 149)** | **Control (n = 151)** | **Unadjusted HR (95% CI)** | **Unadjusted *p* value** | **Adjusted HR (95% CI)** | **Adjusted *p* value** |  | **RIC (n = 281)** | **Control (n = 274)** | **Unadjusted HR (95% CI)** | **Unadjusted *p* value** | **Adjusted HR (95% CI)** | **Adjusted *p* value** | ***p* for  interaction** |
| Ischemic stroke | 22 (14.8%) | 28 (18.5%) | 0.80 (0.46-1.40) | 0.438 | 0.81 (0.46-1.42) | 0.457 |  | 30 (10.7%) | 35 (12.8%) | 0.81 (0.50-1.32) | 0.400 | 0.80 (0.49-1.32) | 0.387 | 0.976 |
| Composite of stroke (ischemic or hemorrhagic),  TIA, or myocardial infarction | 25 (16.8%) | 30 (19.9%) | 0.86 (0.51-1.46) | 0.578 | 0.84 (0.49-1.44) | 0.518 |  | 38 (13.5%) | 52 (19.0%) | 0.68 (0.45-1.03) | 0.066 | 0.67 (0.44-1.02) | 0.064 | 0.488 |
| Stroke (ischemic or hemorrhagic) | 22 (14.8%) | 28 (18.5%) | 0.80 (0.46-1.40) | 0.438 | 0.81 (0.46-1.42) | 0.457 |  | 31 (11.0%) | 36 (13.1%) | 0.82 (0.50-1.32) | 0.403 | 0.81 (0.50-1.31) | 0.386 | 0.966 |
| TIA | 4 (2.7%) | 2 (1.3%) | 2.09 (0.38-11.43) | 0.384 | 2.80 (0.26-29.84) | 0.393 |  | 3 (1.1%) | 8 (2.9%) | 0.36 (0.10-1.37) | 0.119 | 0.41 (0.11-1.61) | 0.203 | 0.097 |
| Myocardial infarction | 4 (2.7%) | 2 (1.3%) | 2.08 (0.38-11.36) | 0.388 | 1.39 (0.21-9.14) | 0.734 |  | 5 (1.8%) | 11 (4.0%) | 0.43 (0.15-1.25) | 0.111 | 0.48 (0.16-1.39) | 0.176 | 0.116 |
| All-cause death | 9 (6.0%) | 9 (6.0%) | 1.02 (0.40-2.56) | 0.975 | 1.05 (0.40-2.71) | 0.927 |  | 12.(4.3%) | 12 (4.4%) | 0.97 (0.43-2.15) | 0.932 | 1.03 (0.46-2.33) | 0.937 | 0.912 |

RIC, remote ischemic conditioning. HR, hazard ratio. CI, confidence internal. TIA, transient ischemic attack.

**Supplemental Table 5. Clinical outcomes after randomization in male and female patients with degree of stenosis ≥ 70% in the per-protocol population**

|  | **Women** | | | | | |  | **Men** | | | | | |  |
| --- | --- | --- | --- | --- | --- | --- | --- | --- | --- | --- | --- | --- | --- | --- |
|  | **RIC (n = 90)** | **Control (n = 105)** | **Unadjusted HR (95% CI)** | **Unadjusted *p* value** | **Adjusted HR (95% CI)** | **Adjusted *p* value** |  | **RIC (n = 183)** | **Control (n = 176)** | **Unadjusted HR (95% CI)** | **Unadjusted *p* value** | **Adjusted HR (95% CI)** | **Adjusted *p* value** | ***p* for  interaction** |
| Ischemic stroke | 21 (23.3%) | 24 (22.9%) | 1.01 (0.56-1.82) | 0.962 | 1.06 (0.57-1.97) | 0.855 |  | 30 (16.4%) | 45 (25.6%) | 0.60 (0.38-0.96) | 0.031 | 0.58 (0.36-0.93) | 0.023 | 0.976 |
| Composite of stroke (ischemic or hemorrhagic),  TIA, or myocardial infarction | 24 (26.7%) | 31 (29.5%) | 0.87 (0.51-1.49) | 0.613 | 0.91 (0.51-1.59) | 0.730 |  | 37 (20.2%) | 57 (32.4%) | 0.58 (0.38-0.87) | 0.009 | 0.55 (0.37-0.84) | 0.006 | 0.488 |
| Stroke (ischemic or hemorrhagic) | 22 (24.4%) | 24 (22.9%) | 1.07 (0.60-1.90) | 0.830 | 1.11 (0.60-2.04) | 0.746 |  | 30 (16.4%) | 46 (26.1%) | 0.59 (0.37-0.94) | 0.023 | 0.57 (0.36-0.91) | 0.017 | 0.966 |
| TIA | 0 (0%) | 4 (3.8%) | - | - | - | - |  | 7 (3.8%) | 7 (4.0%) | 0.93 (0.33-2.65) | 0.890 | 0.84 (0.29-2.45) | 0.749 | 0.097 |
| Myocardial infarction | 2 (2.2%) | 5 (4.8%) | 0.47 (0.09-2.40) | 0.350 | 0.55 (0.07-4.22) | 0.568 |  | 6 (3.3%) | 5 (2.8%) | 1.12 (0.34-3.68) | 0.849 | 1.12 (0.32-3.88) | 0.862 | 0.116 |
| All-cause death | 5 (5.6%) | 4 (3.8%) | 1.45 (0.39-5.40) | 0.579 | 1.53 (0.36-6.51) | 0.569 |  | 7 (3.8%) | 11 (6.2%) | 0.62 (0.24-1.60) | 0.318 | 0.56 (0.21-1.53) | 0.259 | 0.912 |

RIC, remote ischemic conditioning. HR, hazard ratio. CI, confidence internal. TIA, transient ischemic attack. The number of the outcome is too small to calculate HRs or adjusted HRs.

**Supplemental Figure 1. Kaplan-Meier event curve for the other secondary endpoint by sex in the per-protocol population**

(A) Stroke (ischemic or hemorrhagic). (B) Transient ischemic attack. (C) Myocardial infarction. (D) All-cause death.
